# Monitoring of dairy farm management determinants and production performance using structural equation modelling in the Amhara region, Ethiopia

**DOI:** 10.1002/vms3.1140

**Published:** 2023-05-08

**Authors:** Malede Birhan, Yeshambel Mekuriaw, Asaminew Tassew, Firew Tegegne

**Affiliations:** ^1^ Department of Animal Sciences College of Veterinary Medicine and Animal Sciences University of Gondar Gondar Ethiopia; ^2^ Department of Animal Sciences, College of Agriculture and Environmental Sciences Bahir Dar University Bahir Dar Ethiopia

**Keywords:** dairying, management determinants, monitoring, structural equation modelling

## Abstract

**Background:**

Models have been presented to evaluate the link between dairy farm production factors and their degree of association with production determinants. Studies have found causal relationships between production parameters (dairy farm facility, farm hygiene and waste management, feed and nutrition, reproduction performance, health and extension services, mode of transportation, education level and gross revenue) as well as farm efficiency parameters. Furthermore, structural equation modelling (SEM) allows for the estimation of parameters that are not directly quantifiable, known as latent variables.

**Objective:**

The research was designed to identify the dairy management determinants and evaluate farm production performance using an SEM approach in the selected areas of the Amhara region, Ethiopia.

**Methodology:**

In‐person survey using a semi‐structured pre‐tested questionnaire was employed in 2021 to collect primary data on 117 randomly selected commercial dairy producers keeping cross‐breed Holstein Frisian cows in the Amhara region. SEM was used to study the complexity of influences on efficiency measures in milk production utilizing the combined data.

**Results:**

The model result revealed that the relationship between construct reliabilities and farm facilities was significantly varied (*p* < 0.01). The model analysis showed that the level of education has also a positive and statistically significant correlation with the reproduction performance of the dairy farms, (*ρ* = 0.337) and the gross revenue of the farm showed as (*p* = 0.849). Farm gross revenue articulated a positive, strong statistically significant association with feed and nutrition values (*ρ* = 0.906), dairy farm facilities (*ρ* = 0.934), and hygiene and waste management (*ρ* = 0.921). Consequently, the predictors of dairy farm facility's feed and nutrition and hygiene and waste management explained 93.40%, 84.0%, 80.20%, and 88.50% of the variance.

**Conclusion:**

The proposed model was scientifically valid, and training and education have an effect on management practices, subsequently affecting the production performance of the dairy farms.

## Graphical abstract

The research is the first of its kind in the to assess the subject in the study area.

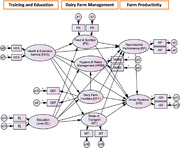

## 1 INTRODUCTION

The food supply has played an important role in the worldwide industry that has contributed significantly to economic growth and rural development in many nations (Camanzi et al., 2018; Mor et al., 2021). India's dairy business has grown significantly over the last three decades, with plans to increase milk output by 9% per year by 2022 (Maini, 2016). The public in developing countries, such as Africa, was concerned about the availability and cost of animal‐derived foods, particularly dairy products such as milk which is unpredictability of consumer demand for feeds and nutrition and the burden of milk price impulsiveness are the major challenges in Ethiopia (Henchion et al., 2017). According to Azage et al. (2013), grass hay and crop leftovers of wheat, barley, teff, and pulse straw are marketed in limited quantities and with poor nutritional values and digestibility of feeds reducing the production capacity and reproductive potential of dairies in Ethiopia.

As a result, to function successfully, dairy producers must focus on improving production variables, such as improving the genetic makeup of dairy cows and improving feed and nutrition requirements (Munyeki & Were, 2017), or improving dairy management practices and dairy farm facilities, which can minimize overall production costs and increase net return, with the lowest total feeding costs and efficient labour utilization (Chen et al., 2015). Furthermore, productive efficiency and feed and nutrition may be the most important factors influencing dairy farm profitability (Knapp et al., 2014). To reduce the complexity of such circumstances, structural equation models containing latent and measurable variables to find out the direct and total effects of the variables might be systematically investigated (Ali et al., 2018). Studies on dairy farm monitoring and adaptive goals toward dairy feed and nutrition are scarce, notably in Ethiopia, and/or rely solely on descriptive statistics (Wondatir et al., 2011). These models, however, did not reflect the net influence of factors on response variables and did not separate the causality pathways that link the cause and effect of the variables directly or indirectly distress the dairy management practices (Drews et al., 2018).

Furthermore, there are also challenges to cultural and religious factors, market inaccessibility and lack of modern transportation, poor health and effective extension service, insufficient feeds and nutrition, dairy farm facilities, farm hygiene and poor management practices, lack of appropriate waste disposal system, and lack of holistic interventions (Mutua, 2018). Despite these obstacles, there are some golden opportunities to boost milk demand and, to a lesser extent, milk consumption trends (van der Lee et al., 2020). To bridge the gap in the causality relationship as well as to find out the direct and indirect effects of the aforementioned measurements on dairy farm management practice is very important. Therefore, the research was designed to measure the dairy farm management determinants and dairy farm production performance using structural equation modelling (SEM) in selected milk sheds of Amhara Region, Ethiopia.

## 2 MATERIALS AND METHODS

### 2.1 Research site description

**FIGURE 1:**
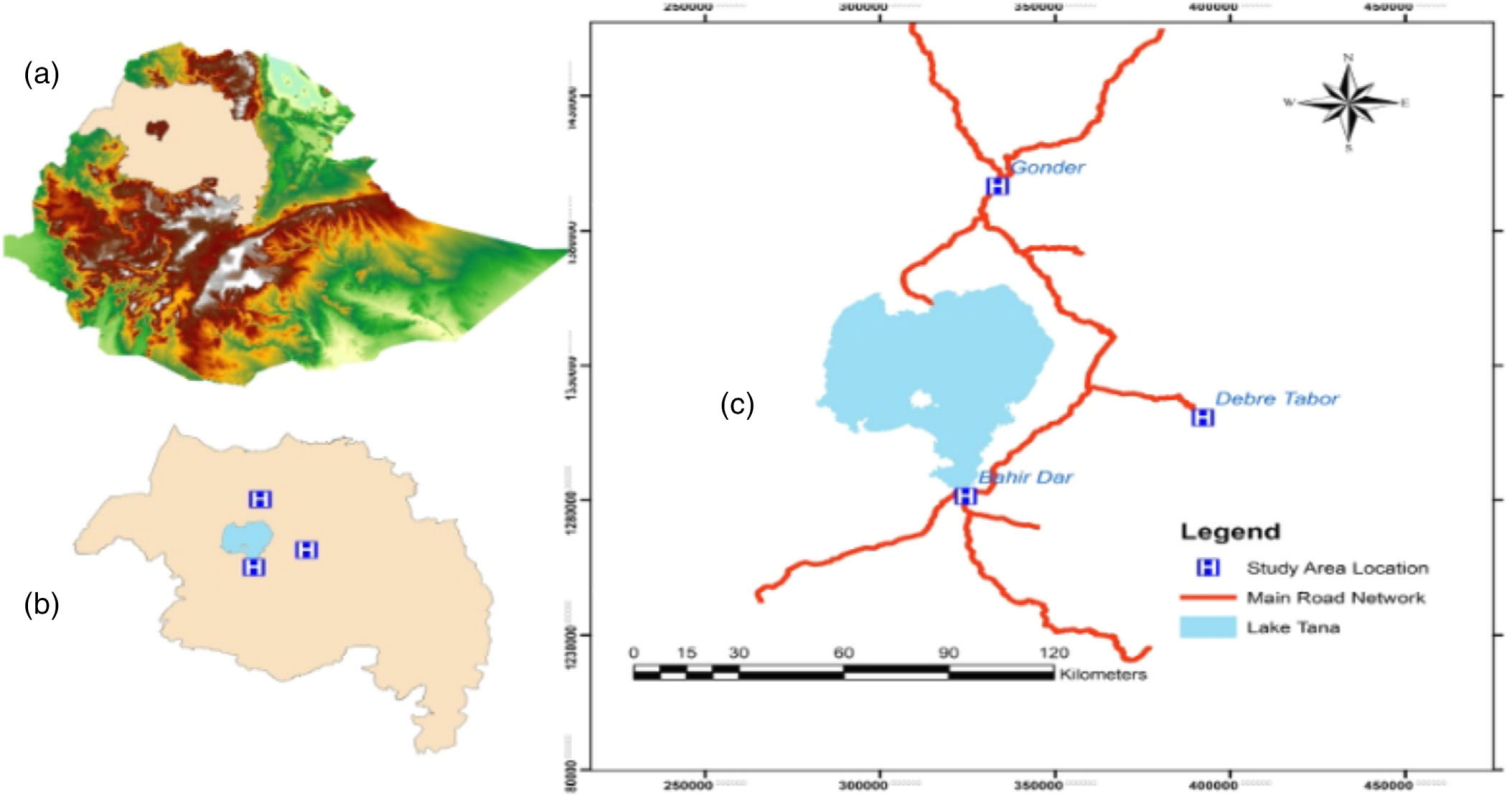
Map of the three research locations in the Amhara region. (A) Map of Ethiopia, (B) map of the Amhara region, and (H) research location in the Amhara region, Ethiopia.

The Amhara region is located between 8^0^45'N and 13^0^45'N latitude and 35^0^46'E and 40^0^25'E longitude in North West of Ethiopia. The survey area is distant from Ethiopia, approximately 578 km far in the Northwest of Addis Ababa. Based on site accessibility and the availability of crossbreed Holstein Frisian dairy cows, the research was conducted on three purposefully selected cities (Bahir Dar, Debre Tabor, and Gondar) with elevated plateaus of 1820, 2706, and 2133 m above sea level and average annual temperatures and rainfall of 20.1, 21 and 23.8°C and 1839, 1270, and 2077 mm correspondingly indicated in Figure 1. The community is Amharic language speaker that is one of Ethiopia's largest federal ethnic divisions, containing the homeland of all people, and its capital is Bahir Dar city. Lake Tana is the country's largest inland water body, which is the source of the Blue Nile River and is located within the region. The region also includes the Semen Mountain National Park, *Ras Dejen*, which is found in the ancient city of Gondar.

### 2.2 Sampling techniques and data collection instruments

To address the specific objective of this research, a total of 864 licensed and legally registered dairy farms have been identified in the Amhara region in the three milk shed areas namely Bahir Dar, Debre Tabor, and Gondar as shown in Figure 1. The farms were categorized as small (*n* = 457), medium, (*n* = 230), and big, (*n* = 177). From the total population of the three categories, 62, 31, and 24 dairy farms were randomly chosen from each category in the dairy farms. Finally, a total of 117 dairy farms (13.54% of the targeted population) were adequately determined based on the sample size computation using a 50% population proportion, 95% confidence interval, and 8.5 margins of error. There is evidence that simple SEM models can be usefully tested even with a small sample size, although the sample size of SEM should be between 100 and 150 to be classified as sample size determination (Anderson & Gerbing, 1988).

A face‐to‐face/in‐person/household survey was used during the primary data collection. As there is limited empirical research on dairy production performance monitoring and management efficiency evaluation using SEM, the data collection instruments are developed by the researchers. The effort demands the development of an empirical framework to assist policymakers in their evaluation of multi‐sources management strategies in the dairy production system of the region. We had control of the sources of variation in the data analysis, we employ dairy farms with similar types of cross‐breeds (Holstein Frisian) cows presented in each respective dairy farm in the three sites. In addition, dairy productivity and reproductive performance were also analyzed based on the individual cow evaluation. To this effect, a closed‐ended questionnaire was pre‐tested and engaged in the research process.

### 2.3 General patterns of SEM analysis

An SEM is a most commonly used approach to causal analysis for variables and constructs that show causality. It distinguishes between latent variables, which are inherently unquantifiable, and measurement variables, which allow the hidden variables to be evaluated. A measurement model is made up of a set of measurement variables that jointly characterize a latent variable (Elmaghraby et al., 2017). The structural model depicts the relationships between the latent variables (*ξ*, *η*), which are represented by arrows connecting them (path coefficients, *γ*). There are two kinds of latent variables: latent cause variables and latent effect variables. Thus, a collection of source variables influences one or more effect variables in an SEM. Furthermore, cause factors must be classified as endogenous (dependent) or exogenous (independent) (Harizanova, 2019). This means that endogenous cause factors are influenced by other latent variables, whereas exogenous variables are not. As a result, the coefficient of determination (*R* ^2^) is determined only for endogenous factors and not for exogenous variables (Krpalkova et al., 2016). Partial least squares structural equation modelling was used to estimate parameters that are not directly quantifiable, known as latent variables. SEM has grown in popularity for multi‐group analysis in agricultural and milk production (Brito et al., 2021).

### 2.4 Data validation and analysis

The validation is carried out using reliability analysis and the exploratory factor analysis method, followed by the confirmatory factor analysis, and finally, SEM was applied to track the performance of the dairy farms in the area (Ravindra et al., 2019). The data gathering took place from Statistical Package for Social Sciences (SPSS) Ver. 23, and analysis of moment structures (AMOS) was applied to examine the data (Kline, 1998).

### 2.5 Construct development and hypotheses formulations

**FIGURE 2:**
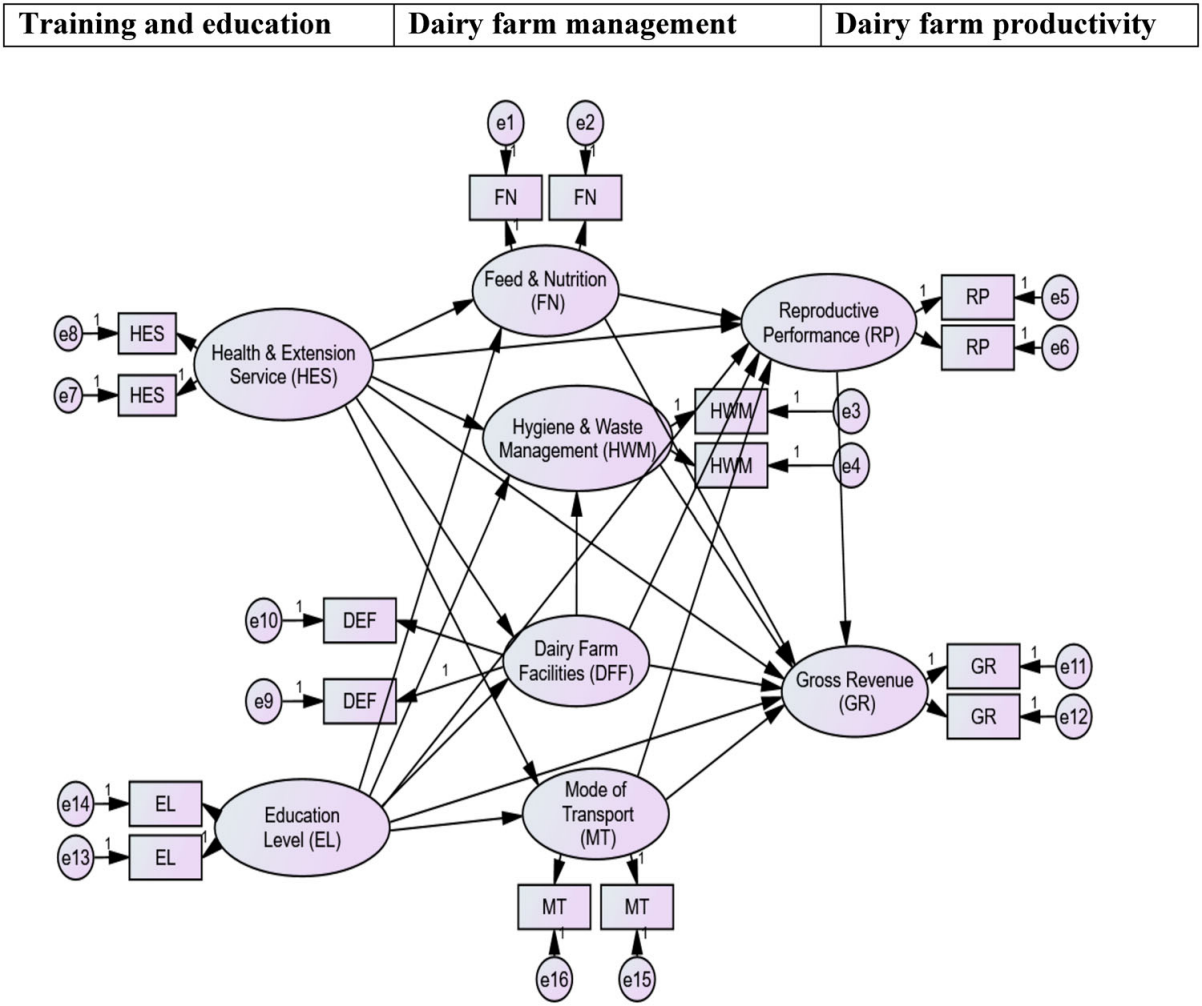
Conceptual framework of the Dairy farm monitoring practice in the Amhara Region DFF, dairy farm facilities; EL education level; FN, feed and nutrition; GR, gross revenue; HES, health and extension service; HWM, hygiene and waste management; MT, mode of transport; RP, reproductive performance.

There is limited empirical research on dairy production performance monitoring and management efficiency evaluation using SEM. The effort demands the development of an empirical framework to assist policymakers in their evaluation of multi‐stage management strategies in the dairy production system of the region. The following predictors were recruited for the analysis used to SEM: educational and training (ED) measured from 1 = reading and writing to 5 having a degree, health care and extension services (HES); measured on a Likert scale, from 1 = strongly disagree to 5 = strongly agree, dairy farm facility (DFF); measured in yes/no, hygienic condition and waste management (HWM); measured in Likert scare, from 1 = strongly disagree to 5 = strongly agree, feed and nutrition (FN); measured in Likert scare, from 1 = strongly disagree to 5 = strongly agree, mode of transportation (MT); measured in yes/no system, reproductive and performances (RP); measured in yes/no, gross revenue (GR); measured as a continuous variable computed daily milk production in litter multiplied by selling price per litter, but it is transformed into a natural logarithm (ln) to make it compatible to the remaining data measurement. After the development of the above constructs and based on the conceptual model presented in Figure 2, the research formulated the following main hypotheses:

H1: The better the training and education level (ED, HES) of the farm owner, the better the dairy farm management (FN, HWM, DFF, and MT).

H2: The better the training and educational status (ED, HES) of a farm owner, the better the productivity of the dairy (RP and GR).

H3: The better the dairy farm management practice of a farm onwer (FN, HWM, DFF, and MT) the better the productivity of the dairy farm (RP and GR).

H4: Dairy farm management (FN, HWM, DFF, and MT) has a mediating effect between farm owner training, education level, and Health and extension service (ED, HES), and farm productivity of the (RP and GR).

Power analyses were also performed to ensure that the required sample size was adequate and that an effect could be seen, given a specified alpha error (0.05), sample size 117, and observed *R* ^2^ of the six endogenous variables. The greater a test's statistical power, the less likely it is to make a Type II error. Power is typically set at 80% or higher (Vangala, 2021), which means that if actual effects are identified in 100 different studies with 80% power, only 80 of 100 statistical tests will identify them. The measured statistical power in this model was 99.999%, which is greater than 80%, indicating that the model is powerful enough to detect significant effects that do exist.

### 2.6 The impacts predictor metrics in dairy farm management

Promoting the use of sustainable dairy farm production through SEM is crucial for fulfilling the expanding population demands of emerging countries (Dellmuth & Tallberg, 2015). To bridge this demand, there is a lack of the necessary technological, organizational, and institutional capabilities (Guadu & Abebaw, 2016). Farmers’ training and education to disseminate improved dairy cattle husbandry practices are an important strategy for increasing dairying's competence and, as a result, adoption (Janetrix, 2019). For this reason, many international aid organizations and national governments advocate large‐scale and ongoing intensive training for farmers in developing nations, but there has been no detailed research on whether these programs are effective or not (Seble et al., 2020). A significant and favourable association exists between dairy farming training and the adoption of higher quality dairy husbandry techniques (Banda et al., 2021). Training and education, according to (Misganaw et al., 2016), induce enhanced yield and boost technical efficiency. Training programs, according to Hundal et al. (2016), have a substantial impact on the adoption of new technologies, aid in the attainment of sustainable dairy production, and, as a result, improve gross income and employment in rural regions. On the other hand, a study emphasizes the value of training, which can help farmers improve their dairy farming skills and generate farm revenues at large (Seble et al., 2020). However, for developing countries like Ethiopia, where livestock productivity (meat and milk) is low despite the large cattle population, the increasing human population combined with increasing demand for animal‐derived food poses a serious challenge. Feed and nutrition management, both in terms of quality and quantity, is one of the most critical causes of the country's low output level (Asredie & Engdaw, 2015). In Ethiopia, the dairy sub‐sector is frequently confronted with feed and nutritional constraints, which are often the most pressing issues and a source of concern in livestock development strategies (Tekeba et al., 2014). According to these sources, during the dry season, ruminants’ basic diets consist of fibrous crop residues and pasture, both of which have low nutritional value, making dairy production difficult. Inadequate energy, protein, and mineral intake, on the other hand, are linked to sub‐optimal dairy cow productivity and reproduction (Sharamo et al., 2021).

Even in normal years, Ethiopia should expect a 35% deficit in feed supply, with this figure rising to 70% during drought years (Derara & Bekuma, 2020). This issue is projected to worsen as the world's population grows and more land is required for crop production. The main causes of dairy feed shortages in Ethiopia have thus decreased grazing pastures as a result of increased arable cropping; the low contribution of improved forage as animal feed (0.25%); and high pricing and inaccessibility of concentrates, which exacerbates the already precarious situation (Marshall et al., 2020).

### 2.7 The role of dairy health and extension service

However, in comparison to the great national potential, the dairy sub‐contribution sector to the national economy is insufficient. The extensive incidence of a range of viral and parasite infections, which considerably reduce dairy cattle output and productivity due to sickness, mortality, and market volatility, is the primary reason for this mismatch (Gizaw et al., 2021). In Ethiopia, animal health extension services include vaccination, modern (clinical services by professionals and paraprofessionals) and traditional treatments, gastro‐intestinal track parasite (deworming) and external parasite (spraying/dipping) controls, disease outbreak investigations and information, herd health advice, and training. Vaccination and contemporary treatments were the most often reported by extension services providers (Bugeza et al., 2017). In general, the quality of animal health care systems is determined by the accessibility, availability, and cost of veterinary services and supplies. Nonetheless, the coverage and access of dairy owners to veterinary services differed significantly across livestock systems, with access being considerably better than other extension services. The most typical problems for health extension services are the relative availability and accessibility of veterinary specialists, basic infrastructure, and other logistics (Gizaw et al., 2019). Most dairy farms’ primary purpose is to enhance earnings. Many farmers are inclined to reduce feed expenses because feed accounts for up to half of all costs on a dairy farm, especially when feed prices are high. Feeding lactation cows, on the other hand, is not a frivolous expense, but rather an investment. Dairy farmers are always looking for feed sources that are less expensive but offer the same results (Baudron et al., 2014).

The high‐producing dairy cow requires a diet that supplies the nutrients needed for high milk production. Carbohydrates, amino acids, fatty acids, minerals, vitamins, and water are all nutrients required by the lactating dairy cow to meet the demand of the mammary gland to produce milk and milk components. However, to produce a cow that will produce a high milk yield, it begins with the nutrition of the calf, lactating cows and heifer (Erickson & Kalscheur, 2020). Dairy cow management interval between drying off and calving, as well as the dry phase, pre‐calving period, and calving, is a period of transition. The management, nutrition, and health practices used during the transition period of a cow's lactation cycle will have a significant impact on cow's productivity and the farm's profitability in the following lactation (Soberon & Van Amburgh, 2017).

### 2.8 Construction validity and reliability

The latent variables were generated after the hypothesized model displayed in Figure 1 was tested and validated to determine how well the model matched the observed data. Feed and nutrition (FN), dairy farm facility (DFF), an education level (ED), dairy cow health and extension service (HES), dairy farm hygiene and waste management (HWM), reproductive performance (RP), and gross revenue are the latent variables in the model (GR). Each of the latent variables was measured in the model using a minimum of four and a maximum of seven observed variables. Analyzing moment structures was used to run and evaluate the model (AMOS‐ver. 21 programs). As the study reveals normality, the maximum likelihood (ML) technique of approximation is used to find the estimates of parameters (Kline, 2011). To examine the model's validity in terms of both model fit (MF) and construct validity (CV), the model was evaluated using Hair et al. (2006). The model's summary results, including the factor loading (FL) and standardized (SFL), the mean (Ӯ), standard deviation (SD) of each latent variable, Cronbach alpha (*α*), chi‐squared test (*χ* ^2^), *p*‐value, the Tucker–Lewis coefficient (TLI), the comparative fit index (CFI), and root mean square error of approximation (RMSEA), are given in Table 1. It is a measure of the internal consistency coefficient, or how closely related a collection of items is as a group, and it discloses the equivalence, homogeneity, and correlation of the statements to assess scale reliability.

**TABLE 1:** The validity and reliability of the constructs

	Measurable variables	Latent variables	FL^*^	SFL	AVE	CR	*Ӯ*	SD	*α* (*ά*)	χ^2^ (*p*‐value)	TLI	CFI	RMSEA
1	Dairy farm facilities				0.832	0.972	1.581	0.414	0.927	83.53 (0.000)	0.77	0.852	0.242
	Water	←‐DFF	1.000	0.884									
	Solar	←‐DFF	0.866	0.765									
	Electricity	←‐DFF	0.835	0.738									
	Toilet	←‐DFF	0.976	0.857									
	Iron roof	←‐DFF	0.910	0.816									
	Brick wall	←‐DFF	0.898	0.793									
	Filed floor	←‐DFF	0.887	0.772									
2	Hygiene and waste management												
	Frequently wash your hands during milking	←‐HWM	1.000	0.922	0.567	0.864	4.177	0.957	0.684 (0.801)	63.07 (0.000)	0.85	0.730	0.266
	The cow udder is frequently washed during milking	←‐HWM	0.702	0.863									
	Milkers have frequently checked the health	←‐HWM	0.650	0.329									
	The milking equipment is usually cleaned and sterilized	←‐HWM	0.720	0.473									
	There is a proper waste‐avoiding system in the farm	←‐HWM	0.635	0.624									
3	Feed and nutrition												
	Frequently used green feed	←‐FN	1.000	0.799	0.675	0.865	4.785	0.958	0.654	17.158 (0.000)	0.747	0.916	0.299
	Frequently used crop residue	←‐FN	0.974	0.962									
	Frequently used commercially formulated feed	←‐FN	0.048	0.036									
	Frequently feed industrial by‐product feeds	←‐FN	0.866	0.842									
4	Reproductive performance												
	Trained heat detection	←‐RP	1.000	0.965	0.73	0.912	1.888	0.2704	0.31 (0.842)	2 (0.002)	0.808	0.936	0.249
	AI delivery record	←‐RP	0.991	0.793									
	AI practice	←‐RP	0.785	0.694									
	AI Payment	←‐RP	0.568	0.577									
5	Health and extension service												
	The extensive services delivery	←‐HES	1.000	0.954	0.549	0.875	4.045	0.995	0.907	9 (0.000)	0.88	0.928	0.192
	Frequently consulted the veterinarian	←‐HES	0.710	0.874									
	Appropriate clinic services	←‐HES	0.824	0.817									
	The animals are regularly vaccinated	←‐HES	0.702	0.879									
	Vaccine service is cheap	←‐HES	0.578	0.672									
	The extension service is provided	←‐HES	0.533	0.541									
6	Mode of transport												
	Transport by ox cart	←‐MT	1.000	0.843	0.645	0.872	1.858	0.806	0.815	2.00 (0.126)	0.956	0.985	0.112
	Transport by foot	←‐MT	0.966	0.948									
	Transport by bicycle	←‐MT	0.570	0.643									
	Transport by vehicle	←‐MT	0.568	0.505									
	Continuous variable												
7	Education in years	EL					0.953	1.310					
8	Gross revenue (GR) (LN transformed)	GR					0.367	7.900					

Abbreviations: AI, artificial insemination; AVE, average variance extracted; CFI, comparative fit index; CR, construct reliability; GFI, goodness of fit index; LN, The nature log is denoted as ln; SFL, standardized factor loading; RMSAE, root mean square approximation; SD, standard deviation; TLI, Tucker–Lewis coefficient index; *α*(*ά*), Cronbach alpha (*α* if item discarded); *χ* ^2^(*p*‐value), Chi‐squared (*p*‐value); Ӯ, Mean.

*All the factor loading (FL) has shown strong statistical significance at *p* < 0.01.

As it is presented in Table 1, the result showed that all the latent variables have exhibited a value of 0.80 or above, which is acceptable (Mor et al., 2019). However, during the test, one of the latent variables, reproductive performance (RP), exhibited a low value (*α* = 0.310) and when two of the items were deleted, the *α* value was improved to *α* = 0.842. Initially, its Cronbach's alpha value was low (0.684), but after revising and when one of the items was discarded with low correlation, the reliability of the Cronbach's improved with a better value of *α* = 0.801. Furthermore, for the sake of additional testing, the average variance extracted (AVE) and construct reliabilities (CR) were computed and given in the same table. All of the latent variables have CR values greater than desirable ≥0.5 limits. Moreover, all of the latent variables have an AVE greater than the cutoff value of 0.5. Both of these tests showed that the model is well fitted to the data.

Furthermore, a common bias method was used to validate the model. Harman's single‐factor and power analysis tests were used to analyze common method bias. The findings imply that common technique bias is not a significant issue in this investigation. According to the unrotated exploratory factor analysis, the average variation explained by the single component is only 34.50% (far below the recommended cutoff of 50%) (Lee & Schatz, 2012). As a result, common method bias is not a problem in our study. Furthermore, power studies were performed to ensure that the required sample size was adequate and that an effect could be seen, given a specified alpha error (0.05), sample size 117, and observed *R* ^2^ of the six endogenous variables (Williams & McGonagle, 2016). The stronger a test's statistical power, the less probable it is to generate a Type II error. Power is normally adjusted at 80% or higher (Vangala, 2021). This indicates that if actual effects are discovered in 100 distinct studies with 80% power, only 80 of 100 statistical tests will identify them. All of the results in this model showed that the observed statistical power is 99.999%, which is greater than 80%, showing that the model is powerful enough to discover significant effects that do exist.

## 3 RESULTS

The purpose of this research is to investigate the role of training and education on the farm management practices and farm productivity of the dairy farm in the Amhara region. The final run was done based on the model's fitting to determine the direct, indirect, and total effects of the various relationships. The overall model test showed that the chi‐square analysis value for the model was 123.408 with degrees of freedom (df = 4) and a *p* of 0.0723, suggesting that the data adequately fit the model. The results presented in Table 2 indicate the correlation matrix between the latent variables. The results showed that all of them exhibited a positive relationship and were statistically significant (*p* < 0.01). For instance, the education level (EL) and DFFs exhibited a positive relationship with a correlation coefficient of *ρ* = 0.728 (*p* < 0.01), revealing that the better education level (EL) has a positive influence on farm owners have a better dairy farm facilities in the respective dairy farms. In line with this, Khan et al. (2018) revealed that the provision of quick and reliable livestock extension services by nongovernmental organizations, high education levels, and good economic resources influenced positively the productivity of dairy farms.

**TABLE 2:** Correlations analysis (*ρ*) among constructs

Measurable parameters	EL	GR	DFF	HES	HWM	FN	RP
GR	0.849**						
DFF	0.728**	0.934**					
HES	0.682**	0.943**	0.961**				
HWM	0.641**	0.921**	0.961**	0.988**			
FN	0.675**	0.906**	0.824**	0.914**	0.899**		
RP	0.337**	0.690**	0.641**	0.799**	0.798**	0.842**	
MT	0.379**	0.738**	0.707**	0.852**	0.851**	0.880**	0.970**

Abbreviations: DFF, dairy farm facilities; EL, education level; FN, feed and nutrition; GR, gross revenue; HES, health and extension service; HWM, hygiene and waste management; MT, mode of transport; RP, reproductive performance.

** Correlation is significant at the 0.01 significant level in (two‐tailed).

Similarly, the availability and accessibility of animal HES in each dairy farm's had a constructive and significantly strong relationship with DFF (*ρ* = 0.961, *p* < 0.01); health and extension service (HES) has also a positive influence on farm owners to have better farm hygiene and waste management (HWM) (*ρ* = 0.988, *p* < 0.01) in the respective dairy farms. Moreover, Omore (2014) described that only around half of dairy farmers have access to preventive measures against animal health services, and it has a great impact on the productivity of dairy farms.

FN has shown a positive relationship with that of HES with a correlation value of *ρ* = 0.914, *p* < 0.01, and mode of transport with that of HES with a correlation value of *ρ* = 0.852, *p* < 0.01. This finding is in line with the works of (VandeHaar, 2006) which reported that excellent feed and nutrition are needed for a cow to express the high potential for dairy production.

Additionally, the model revealed that the level of education has also a positive and statistically significant relationship with the reproduction performance of the dairy farms, with a correlation value of *ρ* = 0.337, *p* < 0.01 and the gross revenue of the farm was shown as *ρ* = 0.849, *p* < 0.01; in addition, the health and extension serveries have a positive and statistically significant relationship with RP (*ρ* = 0.799, *p* < 0.01) and gross revenues (*ρ* = 0.943, *p* < 0.01), which indicates that training and education (HES and EL) have significantly and positively influenced the farm productivity and gross revenue. The other important finding is that the reproductive performance of a dairy farm is also strongly significantly and positively influenced by the farm management practices such as feed and nutrition (FN) (*ρ* = 0.824, *p* < 0.01), DFFs (*ρ* = 0.641, *p* < 0.01), HWM (*ρ* = 0.798, *p* < 0.01), and MT (*ρ* = 0.970, *p* < 0.01). Moreover, farm gross revenue (RG) is also strongly and positively influenced by FN (*ρ* = 0.906, *p* < 0.01), DFF (*ρ* = 0.934, p < 0.01), HWM (*ρ* = 0.921, *p* < 0.01), and MT (*ρ* = 0.738, *p* < 0.01). Therefore, all of these findings suggest that dairy farm management practices have a strong and positive influence on the dairy farm productivity of the study region. In addition, training and education (HES and EL) and dairy farm management practices (HWM, FN, MT, and DFF) have significantly and positively influenced farm productivity and gross revenue. Based on the findings from the result, farm management strategies adopted in the dairy farms such as FN, DFF, HWM, and MT greatly enhanced the farm productivity and gross revenues of the region, and these findings are in line with the findings of other studies (Baudron et al., 2014; Dellmuth, 2015; Munyeki & Were, 2017). Intensive training and education lead to improved farm management techniques, which in turn helps to improve animal health.

### 3.1 Testing the direct relation

The variability of the endogenous variables was investigated while fitting the data with the model. As a result, the *R* ^2^ values are estimated, which represent the variability of the endogenous variables due to their predictor. The *R* ^2^ values for dairy farm facilities, feed and nutrition, hygiene and waste management, mode of transport (MT), reproduction performance (RP), and gross revenue explained in Table 3 are 93.40%, 84.0%, 89.2%, 80.20%, 88.50%, and 97.50%, respectively, indicating that the model explained the outcome values well.

**TABLE 3:** Standardize beta for the direct relationship

Direct effects			Estimate (*β*)	SEM	*p*‐Value	*R* ^2^
DFF	←‐	HES	0.362	0.016	***	0.934 (DFF)
DFF	←‐	EL	0.044	0.012	***	
FN	←‐	HES	0.817	0.057	***	0.840 (FN)
FN	←‐	EL	0.072	0.044	NS	
MT	←‐	HES	0.313	0.019	***	0.802 (MT)
MT	←‐	EL	−0.083	0.014	***	
HWM	←‐	EL	−0.081	0.016	***	0.892 (HWM)
HWM	←‐	HES	0.702	0.049	***	
HWM	←‐	DFF	0.798	0.124	***	
RP	←‐	HES	0.330	0.063	***	0.885 (RP)
RP	←‐	EL	−0.038	0.013	**	
RP	←‐	FN	0.083	0.027	***	
RP	←‐	DFF	−0.910	0.111	***	
RP	←‐	HWM	0.222	0.080	**	
GR	←‐	HES	0.197	0.117	NS	0.975 (GR)
GR	←‐	EL	0.167	0.017	***	
GR	←‐	FN	0.150	0.033	***	
GR	←‐	HWM	−0.070	0.095	NS	
GR	←‐	MT	−0.111	0.132	NS	
GR	←‐	RP	0.210	0.124	NS	
GR	←‐	DFF	0.448	0.205	NS	

*Note*: The overall model showed a Chi‐squared = 123.408, df = 4, and *p*‐value = 0.0723.

Abbreviations: NS, nonsignificant; *p*‐value, significant level; *R* ^2^, regression coefficient; SEM, standard error of the mean.

** Significance level < 0.01.

*** Significant level < 0.001.

Concerning the direct relationship of constructs in the model, it was indicated that except for the path leading EL to FN (*β* = 0.072, *p* > 0.05), all the paths leading from EL to DFF (*β* = 0.044, *p* < 0.001), to HWM (*β* = −0.081, *p* < 0.001), and to MT (*β* = −0.083, *p* < 0.001); HES to FN (*β* = 0.817, *p* < 0.001), to DFF (*β* = 0.362, *p* < 0.001), to HWM (*β* = 0.702, *p* < 0.001), and to MT (*β* = 0.313, *p* < 0.001) were all statistically significant predictors. Therefore, except for the path leading from EL to FN (non‐significant), LE to HWM, and EL to MT (which are unexpectedly negative), all the other path coefficients leading from health and extension service training and education level showed good predictors of the farm management practices (DFF, FN, HWM, and MT) which partially supports H1.

Furthermore, except for the path leading from HES to GR (non‐significant) and EL to RP (unexpectedly negative predictor, *β* = −0.038). The path leading from EL to GR (*β* = 0.017, *p* < 0.001) and HES to RP (*β* = 0.330, *p* < 0.001) was statistically significant predictors of farm productivity (RP and GR), and this partially supported H2.

The third hypothesis is that the greater the dairy farm management practices (feed and nutrition, hygiene and waste management, mode of transport, and dairy farm facilities), the higher the dairy farm productivity and gross revenue. Except for the path leading from feed and nutrition to productive performance (*β* = 0.083, *p*‐value < 0.001 and *β* = 0.150, *p*‐value < 0.001) which partially supported H2, the rest failed to support H3 due to either unexpectedly negative predictors or non‐significant predictors. All these results are similar to the findings of previous studies (Banda et al., 2021; Maini, 2016; Sharamo et al., 2021; Tekeba et al., 2014).

### 3.2 The mediating effect of farm management on‐farm productivity

The research has also hypothesized that dairy farm management enhances farm productivity as a mediating effect for animal health and extension service delivery and education level (EL). In this regard, the total indirect effect presented in Table 4 showed that all of the alternate pathways from animal health and extension service to reproductive performance (*β* = 0.288) and from education level to gross revenue (*β* = 0.041) are statistically and positively significant (*p* < 0.01). All of the pathways from HES to GR (*β* = −0.120, *p* > 0.05) and EL to RP (*β* = −0.052, *p* < 0.01) are statistically non‐significant and unexpectedly negative. Hypothesis H4 partially supported that, “the better the training and education health and extension service, the better the dairy's farm productivity and gross revenue through dairy farm management practice of feed and nutrition, health and extension service, dairy farm facility.” In addition, the total effects of the model revealed that education was a stronger predictor of gross revenue than health and extension service, whereas health and extension service was a greater predictor of reproductive performance. Nonetheless, health and extension service is not a predictor of gross revenue, while education level is a statistically significant predictor of reproductive performance in a negative way, which the researchers did not expect.

**TABLE 4:** Indirect and total effect relationship

Indirect and total effect	Estimate (*β*)	Lower	Upper	*p*‐Value
**Indirect paths (from–to)**				
HES_FN_RP	0.068	0.000	0.134	0.048
HES_HWM_RP	0.156	0.073	0.252	0.01
HES_ DFF _HWM_RP	0.064	0.019	0.142	0.01
EL_FN_RP	0.006	0.000	0.020	0.06
EL_HWM_RP	−0.018	−0.038	−0.007	0.01
EL_ DFF _HWM_RP	0.008	0.002	0.021	0.01
EL_ DFF _RP	−0.040	−0.072	−0.017	0.01
HES_ DFF _GR	0.162	0.067	0.296	0.01
HES_ DFF HWM_RP_GR	0.013	0.000	0.044	0.055
HES_MT_GR	−0.035	−0.181	0.041	0.431
HES_HWM_GR	−0.049	−0.177	0.045	0.207
EL_FN_GR	0.011	−0.002	0.025	0.064
EL_ DFF _HWM_RP_GR	0.002	0.000	0.007	0.037
EL_ DFF _GR	0.020	0.008	0.038	0.01
EL_MT_GR	0.009	−0.011	0.047	0.443
**Total indirect effects**				
TIE_HES_RP	0.288	0.141	0.439	0.01
TIE_EL_RP	−0.052	−0.103	−0.013	0.01
TIE_HES_GR	−0.120	−0.360	0.074	0.208
TIE_EL_GR	0.041	0.013	0.076	0.01
**Total effects**				
TE_HES_RP	0.618	0.446	0.731	0.01
TE_HES_GR	0.077	−0.164	0.295	0.361
TE_EL_RP	−0.090	−0.123	−0.058	0.01
TE_EL_GR	0.209	0.172	0.258	0.01

The final run was done based on the model's findings to determine the direct, indirect, and total effects of the various structures. The chi‐squared analysis value for the variables was 123.408 with four degrees of freedom (df) and a *p* of 0.0723. As a result, the entire model was deemed statistically significant, suggesting that the data were correctly fitted to the model (*p* > 0.05), demonstrating that the data supported the proposed model's distributional assumptions and that the model is accurate. The research model is tested using the path analysis to describe the directed dependencies among a set of variables, and the findings of the results indicated that the data fit the model reasonably well with the goodness of fit index (GFI) of 0.87, confirmatory fit index (CFI) of 0.925, TLI of 0.479, and root mean square of approximate error (RMSAE) of 0.09, but then again low total loading index and Tucker–Lewis coefficient index (TLI) value.

**FIGURE 3:**
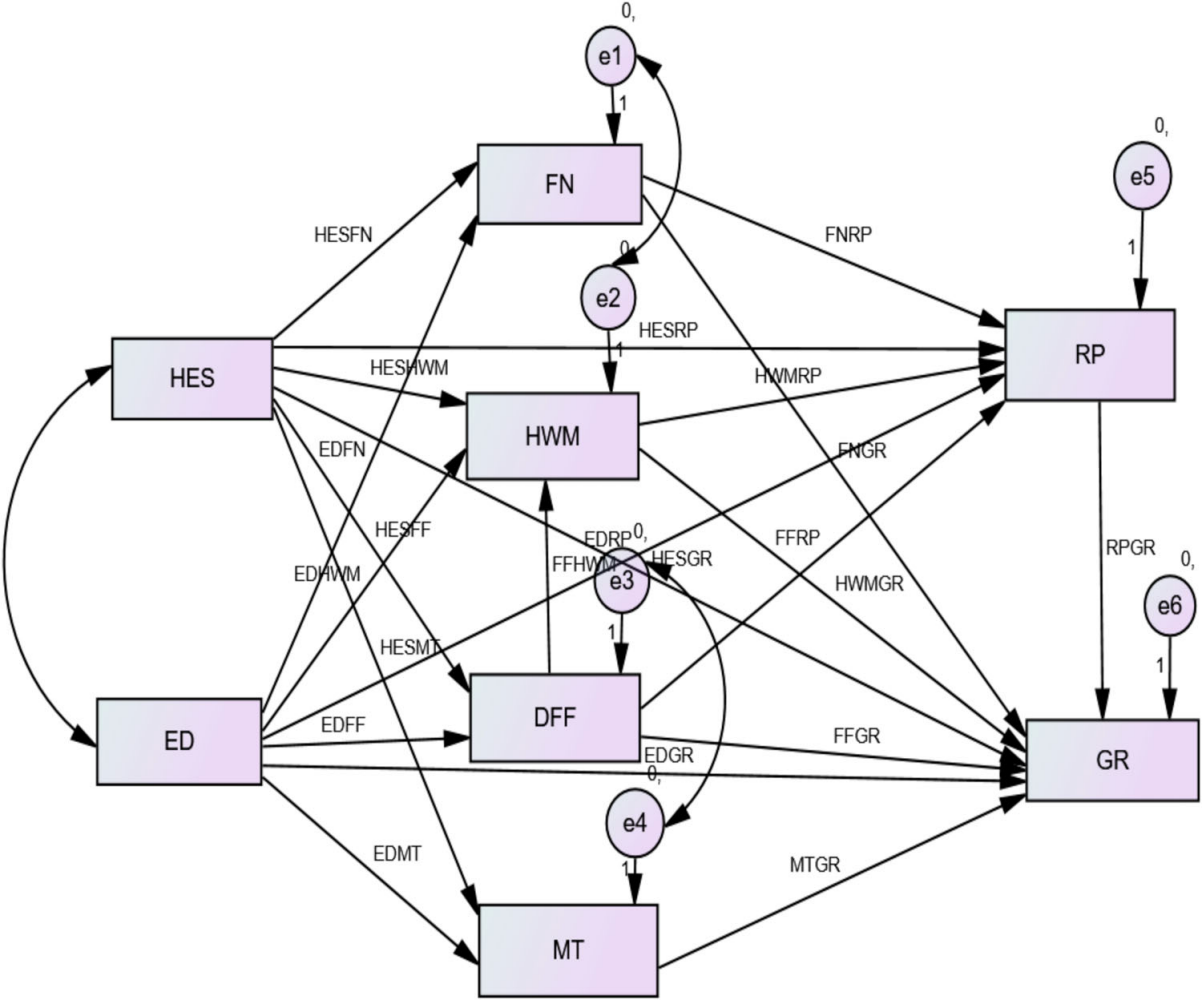
Dairy Farm Monitoring Practice using Structural Equation Modeling (SEM) in the Amhara region FN, feed and nutrition; HES, health and extension service; HWM, hygiene and waste management; RP, reproductive performance; GR, gross revenue; MT, mode of transport; DFF, dairy farm facilities; EL, education level.

The circles indicate latent variables, whereas the rectangles represent measurement variables. Arrows between circles indicate relationships between latent variables (path coefficients analysis). Arrows connecting circles and rectangles indicate the indicator reliabilities of the various measurement variables mentioned above (Figure 3).

Table 2 provides an overview of the latent variables and their associated measurement variables indicating that the model result showed the relationship between construct reliabilities was significant (*p* < 0.01), with a positive relationship between the variables with values of *ρ* = 0.728, hygienic condition and waste management techniques (HWM) of the dairy farm become *ρ* = 0.641, feed and nutrition (FN) of the cows also showed *ρ* = 0.675, and mode of transportation (MT) employed to transfer the dairy products to market place indicated as *ρ* = 0.372. The other variables of the findings revealed that the level of education (ED) has a positively linked and significant association with that of DFF existed in the respective dairy farms. Similarly, the availability and accessibility of health and extension services (HES) in each dairy farm's had a constructive and significantly strong relationship with DFF (*ρ* = 0.961), farm hygiene and waste management (HWM) had a value of(*ρ* = 0.988, feed and nutrition (FN) had a value of *ρ* = 0.914, and mode of transport (MT) had a value of *ρ* = 0.852. Generally, all of these suggests that there is a good and challenging interaction effect between farm management and training and education on HES and ED (DFF, FN, HWM, MT). Additionally, the model revealed that the level of education (ED) has also a positive and statistically significant relationship with the reproduction performance (RP) of the dairy cows, with a probability value of *ρ* = 0.337 and the GR of the farm showed as *ρ* = 0.849. In addition, the health and extension serveries (HES) have a positive and statistically significant relationship with RP (*ρ* = 0.799) and GR (*ρ* = 0.943), which indicates that there is a positive and strong numerical interpretation and significant connotation between farm productivity and training and education on HES and ED as well as RP and GR, which were indicated in Table 2.

The other important variable was the RP of a dairy farm, which is also strongly linked with the farm management practices such as FN (*ρ* = 0.842), DFF (*ρ* = 0.641), HWM (*ρ* = 0.798), and MT (*ρ* = 0.970), through a represented significant relationship. Farm GR shows a positive, strong, and statistically significant association with FN with the value of *ρ* = 0.906, DFF of *ρ* = 0.934, HWM of *ρ* = 0.921, and MT of *ρ* = 0.738. Therefore, these findings suggest that farm management practices have a strong and positive relationship with the dairy farm productivity of the region.

In general, the regression values that qualify as a “good” *R* ^2^ value will vary depending on the situation, with the value becoming 0.5 in the weakest case and greater than 0.5 in the strongest case. The best regression value reading criteria, on the other hand, could be substantially higher, such as 0.9 or advanced. *R* ^2^ values above 0.7 indicate a high level of correlation, whereas *R* ^2^ values below 0.4 indicate a low level of correlation. As a result, the regression values estimated that the predictors of dairy farm facilities (DFF), feed and nutrition (FN), dairy farm hygiene and waste management (HWM), mode of transport (MT), reproduction performance (RP), and gross revenue (GR) explained 93.40%, 84.00%, 80.20%, 89.20%, 88.50%, and 97.50% of the variance, respectively.

The result given in Table 3 indicated that except for the path leading from educational level (ED) to FN (*β* = 0.072), all the path coefficients leading from training and education (ED and HES) showed that good predictors of the farm management system, including ED to DFF (*β* = 0.044), ED to HWM (*β* = −0.081), ED to MT (*β* = −0.083), HES to FN (*β* = 0.817), HES to DFF (*β* = 0.362), HES to HWM (*β* = 0.702), and HES to MT (*β* = 0.313), were all statistically significant predictors partially supporting hypothesis one (H1) that states “The better the training and education (ED, HES) of the farm owner had shown, the better the dairy farm management practices of FN, HWM, DFF, and MT”. However, the negative and statistically significant prediction of ED to HWM and that of MT has indicated an unexpected result.

Furthermore, training and education (HES and ED) were statistically significant predictors of agricultural productivity (PR and GR) based on HES and GR (=0.197); they partially support hypothesis two (H2): The higher the dairy (RP and GR) production, the more training and education a farm owner has received (ED, HES). On the other hand, the negative regression coefficient ( = −0.038) discovered between ED and RP, on the other hand, is unexpected by the research and failed to support H3. The third hypothesis (H3) is that “the greater the dairy farm owner's (FN, HWM, DFF, and MT) management improvement, the higher the dairy farm's production to the (RP and GR)” of the research data presented above (Table 3). Except for the paths leading to FN to RP (=0.083) and DFF to GR (=0.448), which were positive and statistically significant predictors, additional farm management methods are not predictors of the RP and GR. On the other hand, the negative associations between HWM and GR, MT and GR, and DFF and RP, on the other hand, were unexpected and almost failed to support hypothesis three (H3).

After additional investigation, the model's indirect and total effect predictors of training and education (HES and ED) were further evaluated. In this regard, the total indirect effect in Table 3 showed that all channels of health and extension services (HES) through farm management (DFF, FN, HWM, and MT), as well as the quantity of education (ED) the farm owner has through farm management, were investigated (DFF, FN, HWM, and MT). According to Table 4, all of the alternate pathways from HES to RP (*β* = 0.288) and from ED to GR (*β* = 0.041) are statistically and positively significant. All of the pathways from HES to GR (*β* = −0.120) are statistically significant and unexpectedly negative. All of the lines from ED to RP (*β* = −0.052) are unexpectedly negative, but not significant. Hypothesis four (H4) was partially supported: “The better the training and education (ED, HES), the better the dairy's productivity (RP and GR) through dairy farm management (FN, HWM, DFF, and MT).” In addition, the overall effects of the model revealed that education (ED) was a stronger predictor of GR than HES, whereas HES was a greater predictor of PR. Nonetheless, HES is not a predictor of GR, while ED is a statistically significant predictor of PR in a negative way, which the researchers did not expect.

## 4 DISCUSSIONS

All predictor variables’ estimated factor loadings (FL) produced as a regression weight in the AMOS were statistically strongly significant (*p* < 0.01), and the regression weight was found to be above 0.50, implying convergent validity (Ali et al., 2018). According to (Yu & Xie, 2012), the standardized regression weights should be 0.50 or higher, preferably 0.7 or higher. They also suggested that cutoff values such as RMSEA 0.06, TLI > 0.95, CFI > 0.95, and CFI > 0.95 were appropriate for categorical outcomes. Except for the built MT, the TLI value of the majority of the constructs is less than 0.95. Concerning TLI, except for DFF and HWM, it exhibits a good fit with TLI values greater than 0.9. HWM has the lower TLI, which is 0.55. Furthermore, the farm management practices implemented in the dairy farm such as FN, DFF, HWM and MT required strongly promoted farm productivity (PR and GR), and these findings are highly relevant to the findings of Soteriades et al. (2016). Furthermore, training and education for dairy farm owners, such as health and extension services (HES) and educational (ED), are both good predictors of farm management systems (HWM, DFF, MT) and farm production (RP and GR), according to the data (Soteriades et al., 2016). Furthermore, farm management methods (HWM, FN, and DFF) are good predictors of one of the farm's productions (RP), which supports the findings of Drews et al. (2018). As shown in Table 4, there was a highly significant difference (*p* < 0.001) in the availability of dairy farm infrastructure, dairy cow health and extension service delivery, farm hygienic condition and waste management practices, and farm reproductive performance. The model analysis revealed a substantial difference (*p* < 0.01) between reproductive performance and artificial insemination services, as well as diet and nutrition requirements, dairy cow health, and extension services.

## 5 CONCLUSIONS

The educational levels of dairy farm operators were also demonstrated to have a significant impact on farm hygiene and waste management procedures, and the relationship between the mode of dairy product transportation similarly affected the dairy management practice. The dairy cow health status and extension service providers also presented substantial transformation on the management of dairy farm hygienic conditions and farm waste controlling strategies in the study areas, whereas the feed and nutritional requirements of the dairy farm had a significant impact on the educational levels of the dairy actors. As a consequence of these findings, the dairy farm management practices mainly focusing on FN, DFF, HWM, and MT, as well as farm productivity and reproductive enactments (RP and GR) of the dairy farm, were positively and significantly influenced by access to training and education level of the dairying. The findings of the research are based on the application of the model only in the selected studied areas, and its use in other areas needs due consideration of the variability of the dairy farms in the specific location.

## AUTHOR CONTRIBUTIONS

*Resources and supervision*: Yeshambel Mekuriaw. *Supervision*: Asaminew Tassew and Firew Tegegne.

## CONFLICTS OF INTEREST STATEMENT

The authors declare no conflict of interest.

## ETHICS STATEMENT

The research does not include any human or animal participants.

## Funding information

Since this is my PhD dissertation work, I did not receive any financial support or funding for this research.

### PEER REVIEW

The peer review history for this article is available at https://publons.com/publon/10.1002/vms3.1140.

## Data Availability

The data that support the findings of this study are available on request from the corresponding author. The data are not publicly available due to privacy or ethical restrictions.
